# Mediterranean Diet and Lifestyle in Persons with Mild to Moderate Alzheimer’s Disease

**DOI:** 10.3390/nu16193421

**Published:** 2024-10-09

**Authors:** Ligia J. Dominguez, Nicola Veronese, Angela Parisi, Flavia Seminara, Laura Vernuccio, Giuseppina Catanese, Mario Barbagallo

**Affiliations:** 1 Department of Medicine and Surgery, University Kore of Enna, 94100 Enna, Italy; 2 Geriatric Unit, Department of Internal Medicine and Geriatrics, University of Palermo, 90144 Palermo, Italy; nicola.veronese@unipa.it (N.V.); angelaparisi89@libero.it (A.P.); semifla94@gmail.com (F.S.); lvernuccio@virgilio.it (L.V.); giuseppina.catanese@policlinico.pa.it (G.C.); mario.barbagallo@unipa.it (M.B.)

**Keywords:** Alzheimer’s disease, dementia, mediterranean diet, aging, malnutrition, physical activity

## Abstract

Due to the continuous aging of the population and consequent increase in dementia, focus on its prevention is of growing importance for public health. Since effective pharmacological treatments are not yet available, other determinants of cognitive decline have become fundamental. Several studies have indicated that the Mediterranean diet (MedDiet) is associated with reduced incident cognitive decline and dementia, but few studies have been conducted in persons already diagnosed with Alzheimer’s disease (AD). We age-matched 73 patients with mild–moderate AD with 73 controls (mean age for the whole group = 76.5 ± 6.5; 67.5% women). The cases had a significantly lower adherence to the MedDiet and lower physical activity vs. controls, where only one participant (1.4%) had a high adherence to the MedDiet among cases compared to 5.5% among controls, while 52.5% of the cases had a moderate adherence to the MedDiet vs. 82.2% in controls. In multivariate analysis, only the presence of AD was significantly associated with a lower adherence to the MedDiet vs. controls. Other factors examined (gender, age, physical activity level, multimorbidity, and polypharmacy) were not significantly associated with adherence to the MedDiet. Thus, AD patients had a low adherence to the MedDiet and very low physical activity. Public health strategies aimed at promoting the Mediterranean diet and physical activity for older people should be a priority.

## 1. Introduction

The world is continuously aging. Currently, most humans around the world can expect to live beyond the age of 60 [1]. Consequently, the prevention and treatment of age-related chronic diseases, such as neurodegenerative diseases, are of growing importance for public health [2,3]. It is estimated that the incidence of dementia almost doubles every 5 years after the age of 65. By the time people reach their nineties, almost one-third of adults will meet the diagnostic criteria for dementia. In 2019, it was estimated that 57 million people had dementia worldwide, and the projection for 2050 has increased to 153 million [4]. The most diagnosed forms/types of dementia are Alzheimer’s disease (AD), the vascular form, and the mixed form. By causing progressive cognitive and functional deterioration, they represent major causes of disability [5].

Aging is one of the greatest risk factors for developing dementia, as with other chronic conditions [6]. By the time adults are in their thirties, fundamental cognitive abilities, such as episodic memory, reasoning, processing speed, and spatial visualization, begin to decline [7]. Regardless of other factors, “biological” aging is in itself accompanied by a systemic pro-inflammatory response and modifications in the central nervous system, including brain atrophy, white matter degradation, and neuropathological protein accumulation [8], which increases susceptibility to the development of neurodegenerative diseases. However, further investigations are needed to determine which additional factors or pathophysiological mechanisms lead some persons to develop a neurodegenerative disease while others will remain “resilient” to any form of dementia [9].

Other factors may contribute to the onset of cognitive decline, including genetic predisposition and socioeconomic and environmental influences such as diet and physical activity [10]. Since effective pharmacological treatments are not yet available to treat cognitive deterioration and dementia [11], prevention becomes increasingly fundamental [12]. Certain nutritional regimes and specific foods, especially if associated with a healthy lifestyle that includes physical activity and regular circadian rhythm, have long been studied as possible modifiable factors capable of delaying the onset and severity of cognitive decline [12]. Several studies have indicated the Mediterranean diet (MedDiet), or rather the “Mediterranean lifestyle”, as having significant features associated with a reduced incidence of various non-communicable diseases, including dementia [12,13,14,15,16].

Nutritional problems such as weight loss, lack of appetite, and sarcopenia are frequent among patients suffering from cognitive decline, especially in the more advanced stages of the disease [17]. These correlate with relevant adverse events, including institutionalization, morbidity, and mortality [18]. Some studies have also shown that in patients with early-stage AD or mild cognitive decline, nutritional alterations increase the risk of disease progression to a more severe stage [19,20] and of behavioral and psychiatric symptoms [21]. In the early stages of the disease, memory and concentration deficits can affect planning, shopping, and preparing meals. As cognitive deterioration progresses, eating adequately becomes an increasingly difficult challenge: sensory alterations (e.g., reduction in smell and taste), loss of appetite (senile anorexia), difficulty in communicating discomfort or sensation (e.g., hunger, thirst, tiredness, pain, constipation), behavioral disorders (e.g., aggression, apathy, depression, wandering), and in the last stages, the possible onset of dysphagia. These factors increase the risk of malnutrition, complications, and disease progression [17].

Based on this background, we aimed to evaluate the adherence to the MedDiet and lifestyle of persons affected by mild to moderate AD and its association with the severity of the disease as well as with anthropometric parameters, self-efficacy, comorbidity, polypharmacy, and physical activity in comparison with age-matched control participants without cognitive issues. We also evaluated the consumption of specific foods that have been shown to have neuroprotective properties.

## 2. Participants and Methods

### 2.1. Participants

In the present cross-sectional study, older men and women undergoing an evaluation at the Cognitive Disorders and Dementia (CDCD) ambulatory clinics of the Geriatrics Section of the “Azienda Ospedaliera Universitaria Policlinico ‘Paolo Giaccone’” in Palermo, Italy, were consecutively enrolled from 1 February 2023 to 1 August 2024. All patients lived at home in an urban setting with their relatives or caregivers, and none of them lived alone or were institutionalized. Inclusion criteria were (1) age > 60 years; (2) diagnosis of AD (mild to moderate), according to DSM-5 criteria [22]. In brief, this includes significant cognitive decline from a previous level of performance in one or more cognitive domains referred by the patient, a knowledgeable informant, or the clinician; a substantial impairment in cognitive performance in standardized neuropsychological testing; the cognitive deficits interfere with independence in everyday activities; exclusion of delirium or other mental disorder. In addition, all the participants included retained a minimum degree of autonomy and self-sufficiency; therefore, they were able to carry out regular physical activity (even a simple daily walk) and could consume different types of food as they were free from problems that prevented their intake (e.g., dysphagia, total edentulism, etc.). On the contrary, we excluded patients without a complete evaluation of functional and cognitive status or a cognitive decline due to a primary psychiatric disorder such as schizophrenia or bipolar disorder. The participants in the study were recruited based on the voluntary acceptance by the patient or caregiver to be part of the study and interviewed via a questionnaire that included various parameters (anthropometric, nutritional, and geriatric assessments) as described below. The participants in the control group were identified among outpatients attending the osteoporosis ambulatory clinic of the same University Hospital; in particular, autonomous and self-sufficient persons not affected by cognitive decline were enrolled. Written informed consent was obtained from all participants involved in the study, and in case of the patient’s inability, the legally authorized delegate provided informed consent. The study was conducted in accordance with the Declaration of Helsinki on the ethical principles for medical research involving human subjects [23].

All data analyzed in the present study were obtained as part of routine evaluation, diagnosis, and treatment. In accordance with the current Italian law (Gazzetta Ufficiale della Repubblica Italiana, Serie Generale n. 76 del 31 May 2008), we acknowledge our Institution’s Ethical Committee (Comitato Etico Palermo 1 A.O.U.P. ‘P. Giaccone’) (protocol n. 22 del 3 September 2024) about this observational research regarding usual clinical practice by sending an official letter.

### 2.2. Outcome: Adherence to the Mediterranean Diet

The degree of adherence to the MedDiet was assessed through the MEDAS (*Mediterranean Diet Adherence Screener*) [24], a questionnaire initially developed during the PREDIMED study (*Prevención con Dieta Mediterránea*) [25] and consisting of 14 items, which are quick and easy to administer. Briefly, each question was given a score of “0” or “1”. One point was awarded for the consumption of olive oil as the main source of cooking fats (question 1); 4 or more tablespoons of olive oil per day (question 2); 2 or more portions of vegetables per day (question 3); 3 or more servings of fruit per day (question 4); less than 1 serving of red meat or sausage per day (question 5); less than one serving of animal fats per day (question 6); less than 1 carbonated or sweetened drink per day (question 7); 7 or more glasses of wine per week (question 8); more than 2 portions of legumes per week (question no. 9); 3 or more portions per week of fish or seafood (question 10); less than 3 times a week of commercial pastry (question no. 11); 3 or more times a week of dried fruit (question no. 12); chicken, turkey, or rabbit instead of beef, pork, and cured meats (question n.13); and 2 or more times a week of boiled vegetables, pasta, or rice seasoned and sautéed (question no. 14). The MEDAS score ranges from 0 to 14 points. Even if no definitive cut-offs exist, we used, for the purposes of this work, a score less than 6 to indicate low adherence to the MedDiet, a score between 7 and 10 as intermediate adherence, and a score between 11 and 14 considered as high adherence [25].

### 2.3. Demographics

Other than age and sex, we included physical activity level carried out in the last seven days through the IPAQ SHORT FORM (*International Physical Activity Questionnaire*) [26], which differentiates between heavy activity (e.g., heavy lifting, aerobics, digging, or fast bicycling…), moderate activity (lifting light weights, doubles tennis, cycling at a moderate pace…), or simply walking (of at least 10 min), as well as time spent sitting on weekdays (e.g., sitting at a desk, reading, visiting friends, lying down to watch television, or sitting), indicating for each duration and weekly frequency; smoking status, categorized in no, previous, or current.

### 2.4. Multidimensional Evaluation

A multidimensional evaluation was performed on all patients, investigating the ability to perform activities of daily living (ADL), which defines the level of dependence/independence in six activities (bathing, feeding, toileting, dressing, transferring in and out of bed or chair, urine, and bowel continence) ranging from zero to six [27,28]; Instrumental Activities of Daily Living (IADL) considering eight activities, which are more demanding cognitively and physically with respect to ADL, i.e., using the telephone, managing finances, shopping, taking medications, preparing meals, using transportation, doing housework, and washing [29,30]; the global degree of cognitive decline assessed with the Mini-Mental State Examination (MMSE) considering seven different cognitive areas and 30 different questions divided into orientation over time, orientation in space, word recording, attention and calculation, evocation, language, and constructive praxis. Multimorbidity was considered when a participant had a diagnosis of two or more medical conditions [31] commonly present in older people, such as heart attack, hypertension, hypercholesterolemia, stroke, diabetes, chronic lung disease, arthritis, asthma, osteoporosis, gastric or duodenal ulcer, cancer, hip fracture, and cataracts. Polypharmacy is defined as the regular use of over five medications [32].

### 2.5. Nutritional Evaluation

Weight and height were recorded by trained residents in geriatric medicine. Body mass index (BMI) was recorded classifying participants into underweight (BMI < 19 kg/m^2^), normal weight (BMI between 19.1 and 25 kg/m^2^), overweight (BMI between 25.1 and 30 kg/m^2^), and obese (BMI > 30 kg/m^2^). We also recorded data about the dietary intakes of some specific nutrients that have been associated with beneficial or harmful effects on cognition, such as alcohol drinking, spices, tea, coffee, cocoa, red fruits, and salt [33].

### 2.6. Statistical Analysis

We used mean and standard deviation (SD) to describe continuous variables and percentages for categorical variables. Baseline characteristics of the study participants were compared between cases and controls, using an independent *T*-test for continuous variables and Chi-squared for categorical variables. We categorized the adherence to the MedDiet as moderate-to-high vs. low since only one case reported a high adherence to the MedDiet. The association between the adherence to the MedDiet and factors significantly different between cases and controls (*p* < 0.05) was explored using a logistic regression analysis. The factors potentially associated with the outcome of interest were based on the previous literature about risk factors of poor adherence to the MedDiet [34]. The results are reported as odds ratios (ORs) and their 95% confidence intervals (CIs). Finally, in people affected by AD, we explored the association between MEDAS and the domains of multidimensional evaluation, using simple correlation analysis and reporting data as rho and *p*-values due to a non-parametric distribution of the MEDAS. All statistical analyses were carried out using SPSS software version 26.0, were two-tailed, and a *p*-value < 0.05 was considered statistically significant.

## 3. Results

In this study, we matched 73 patients with mild to moderate AD (cases) with 73 controls for age. The mean age of the whole group was 76.5 ± 6.5, and 67.5% were women. As shown in Table 1, the cases did not differ in terms of age (*p* = 0.69), but they were more frequently males. The cases reported significantly lower moderate (*p* = 0.03) and low (*p* < 0.0001) physical activity levels compared to the controls. The cases reported an important impairment in ADL (mean = 3.4) and IADL (mean = 1.9) as well as in MMSE (mean = 16.7/30). As expected, multimorbidity and polypharmacy were more frequent among the cases compared to the controls (Table 1).

**Figure 1:**
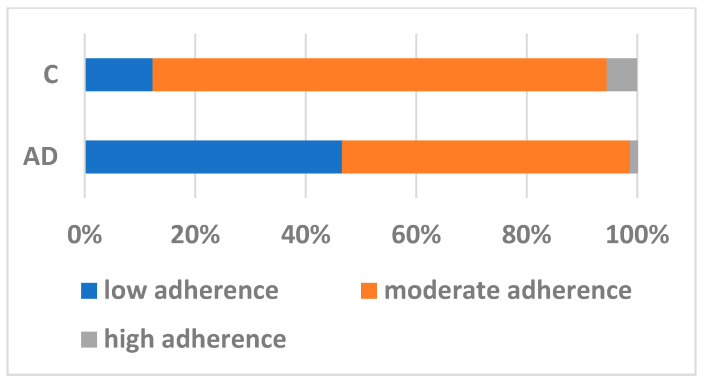
Adherence to Mediterranean diet in cases and controls. AD: Alzheimer’s disease; C: controls.

About nutritional information, the cases reported less frequently the use of spices (*p* = 0.04) and coffee (*p* < 0.0001) but more frequently the consumption of red fruits and salt. As shown in Table 1, the cases reported significantly lower adherence to the MedDiet in the mean of 1.4 points. In Figure 1, data from the differences in the degree of adherence to the MedDiet between the cases and controls are shown: very few (1.4%) had high adherence to the MedDiet among the cases compared to 5.5% among the controls; 52.5% of the cases had a moderate adherence to the MedDiet compared to 82.2% in the control group (*p* < 0.0001 for these comparisons).

Table 2 shows the multivariate analysis, taking as an outcome the moderate-to-high adherence to the MedDiet and as factors all the factors significantly different among the cases and controls. Only the presence of AD was associated with a lower adherence to the MedDiet compared to the controls (OR = 0.222; 95% CI: 0.058–0.848; *p* = 0.028), whilst the other factors examined (gender, age, physical activity level, multimorbidity, polypharmacy, the consumption of red fruits, more than 3 times in a week, coffee consumption, and the use of salt) reported a *p*-value > 0.20.

**Figure 2:**
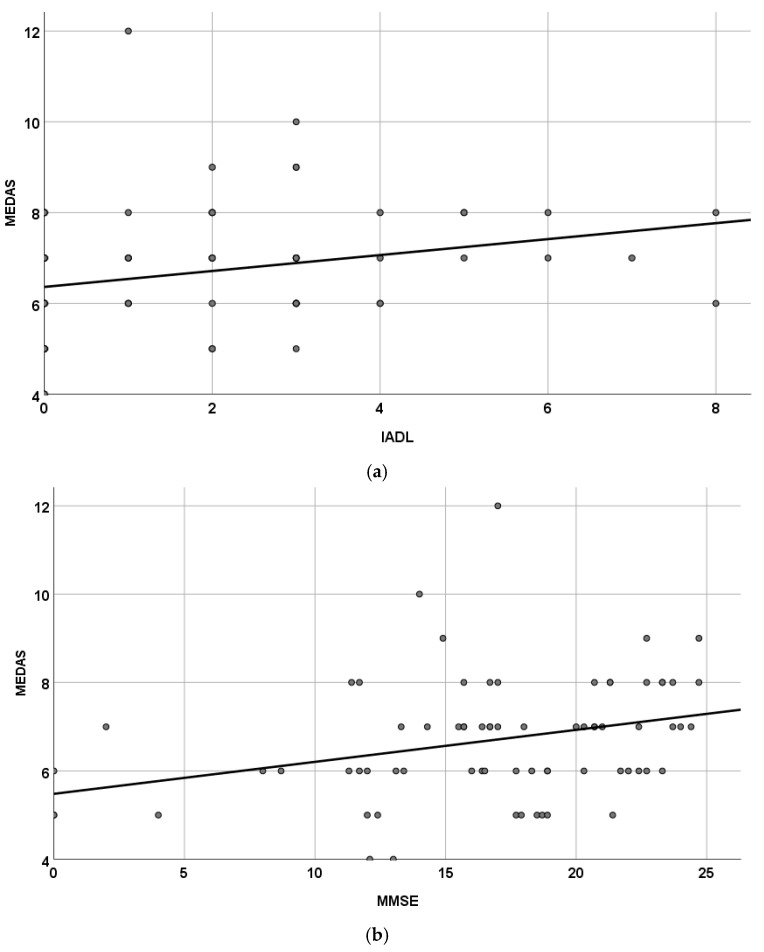
Correlations between adherence to the Mediterranean diet and instrumental activities of daily living (**a**) and with mini-mental state examination (**b**). IADL: Instrumental Activities of Daily Living); MEDAS: Mediterranean Diet Adherence Screener; MMSE: Mini-Mental State Examination.

Finally, we analyzed the correlations between the adherence to the MedDiet and the domains of multidimensional evaluation. Considering these domains, only IADL (rho = 0.334, *p* < 0.001) and MMSE scores (rho = 0.331, *p* < 0.001) were associated with MEDAS, as graphically reported in Figure 2.

## 4. Discussion

In the present study, among 146 participants examined, those with a diagnosis of mild to moderate AD had significantly lower adherence to the MedDiet pattern assessed with the MEDAS questionnaire compared to those participants who were cognitively competent. Practically none of the participants with dementia had a high adherence to this high-quality dietary pattern, while most of the controls (>80%) had an intermediate good adherence in this Mediterranean population. Likewise, the level of physical activity carried out in the last seven days assessed with the IPAQ SHORT FORM was also remarkably low among dementia patients, with almost half (45%) not carrying out any physical activity vs. almost all (93%) of cognitively competent patients habitually carrying out at least light physical activity and 14% moderate physical activity. Few participants from both groups used to smoke or had a habitual consumption of alcoholic beverages (other than red wine); both parameters were comparable for both groups. The consumption of certain foods considered neuroprotective resulted slightly different between the two groups, with cases reporting less frequently the use of spices and coffee but more frequently consumption of red fruits and salt. Nevertheless, all participants had a low average consumption of coffee (two cups per day), below the general recommendation of 3–5 cups per day [35]. We found significant differences between the two groups regarding comorbidity and pharmacological history, with most participants with dementia having more than three pathologies and taking over five medicaments per day vs. lower numbers for controls. In multivariate analysis only the presence of AD was significantly associated with a lower adherence to the MedDiet compared to controls. Other factors examined (gender, age, physical activity level, multimorbidity, and polypharmacy) were not significant. There was a positive significant association between adherence to the MedDiet and cognition assessed with the MMSE score as well as with self-autonomy as an ability to perform IADL.

Although there are several studies specifically investigating the role of the MedDiet in the prevention of cognitive decline and dementia that have found that this high-quality dietary pattern seems to be protective for the development of dementia [12,13,14,15,16], this is one of the few studies exploring the adherence to the MedDiet in patients with a clinical diagnosis of AD. Several systematic reviews and meta-analyses have explored the association of adherence to the MedDiet with cognitive decline and/or incident dementia [13], and most of them have shown an inverse association of a higher adherence to the MedDiet with a slower cognitive decline and/or reduced progression to AD. There are also conflicting results regarding the efficacy of the MedDiet for age-related cognitive function [36,37]. In an umbrella review, out of 11 meta-analyses, 3 reported convincing evidence of benefit, 2 were highly suggestive of benefit, 3 were suggestive of benefit, 1 reported weak evidence, and only 2 reported no evidence [38]. Disparity of neuropsychological assessment methods used appeared to be a plausible contributor to the lack of consensus among study findings. Other diets very similar to MedDiet, such as the Mediterranean dietary approaches to stop hypertension intervention for neurodegenerative delay (MIND) diet, have shown positive results [39,40,41,42]. A large meta-analysis of three cohort studies among over 200,000 participants reported that increased adherence to the MIND diet was significantly associated with a decreased risk of dementia [39]. Likewise, a US cohort study of older adults with a baseline mean age of 84.2 years showed that the MIND and the MedDiet patterns, in particular the consumption of green leafy vegetables, were inversely associated with amyloid beta load, phosphorylated tau tangles, and global AD pathology at post mortem analyses [40]. A recent 6-month pilot randomized controlled trial (RCT) was conducted among 93 participants with prodromal AD in four European countries with 3 intervention arms: (1) multimodal lifestyle intervention (nutritional guidance, exercise, cognitive training, vascular/metabolic risk management, and social stimulation); (2) multimodal lifestyle intervention + medical food product; and (3) regular health advice (control group). Adherence to dietary advice was assessed with the Healthy Diet Index and MEDAS. Dietary quality in the intervention groups improved in this population with prodromal AD. Nutrient intakes remained unchanged in the intervention groups, while the control group showed a decreasing nutrient density [43]. These preliminary results suggest that dietary intervention as part of multimodal lifestyle interventions is feasible in this type of patient and that further studies are needed to confirm these encouraging results. There is also evidence of an inverse significant association between adherence to the MedDiet and other relevant mental health conditions, in particular depression [44].

We have considered some foods for which there is some evidence of neuroprotection or neurodamage that were available in the questionnaire. For example, red fruits (berries), which are rich in potent antioxidants such as flavonoids and anthocyanins, have shown neuroprotective effects, but most of the evidence comes from experimental studies in animal models or cellular cultures [45]. There are few intervention studies in humans. A systematic review of studies exploring anthocyanin consumption (i.e., berry juices) and cognitive outcomes in humans found that 6 of 7 studies reported improvements in single or multiple cognitive outcomes. However, there were important methodological limitations because most were small trials with high heterogeneity [46]. Coffee is currently considered helpful in many aspects [47]. However, in terms of neuroprotection, there is no consistency in the literature on the association of the positive effects of coffee on long-term cognition [48,49]. A recent meta-analysis indicated that limited (1–4 cups/day) daily coffee consumption reduces the risk of AD, whereas excessive consumption (>4 cups/day) might increase the risk [49]. A recent meta-analysis reported a linear association between tea intake and risk of dementia, with a significantly decreased risk of dementia for each 1 cup per day increase in tea consumption [50]. Alcohol consumption has contradictory evidence; a small amount, less for women, is reported to be protective [51], but some studies that inhibit any consumption of alcohol state that it is especially harmful to the brain [52]. Preliminary evidence showed some neuroprotective effects of cocoa consumption [53] but there are still debated results about its potential use in the prevention of cognitive decline and dementia [54]. Likewise, there is no consensus on the indication of the consumption of dietary spices for neuroprotection [55]. A recent Mendelian randomization study exploring the causal relationship between dietary salt intake and dementia risk found strong evidence of this association [56]. We found a significantly lower consumption of spices and coffee in AD patients vs. controls and a higher consumption of red fruits and salt in these patients. However, none of them were significantly associated with the multivariate analysis.

There is also evidence showing that combining a healthy diet with a healthy lifestyle is associated with a reduced risk of developing dementia [3,41,57,58,59]. In this regard, a recent remarkable study using data from 586 deceased participants of the Rush Memory and Aging Project who were followed for up to 24 years with data on cognitive testing and lifestyle factors collected close to death and a complete neuropathologic autoptic evaluation found that a higher lifestyle score was associated with better global cognitive functioning close to death; in multivariable-adjusted models, a one-point increase in lifestyle score was significantly associated with higher global cognitive scores. Even when including common dementia-related brain pathologies in the multivariable-adjusted models the strength and significance of the association was maintained. A higher lifestyle score was associated with lower beta-amyloid load in the brain. Thus, a healthy lifestyle may afford a cognitive reserve in older adults, contributing to the maintenance of cognitive abilities independently of the presence of dementia neuropathologies [41].

There are fewer studies considering the role of nutrition in the initial stages of AD in clinical settings. Due to the lack of an efficacious pharmacological treatment, this evidence is relevant in order to target modifiable nutritional factors that potentially may help prevent or delay further cognitive deterioration. Former evidence showed that malnourished patients with AD display a more rapid cognitive deterioration and progression within one year when compared with well-nourished patients [60,61]. This has been confirmed by a recent study showing that participants with a less healthy diet and worse nutritional status were associated with a higher risk of cognitive decline clinical progression in a sample of patients with subjective cognitive disease, mild cognitive impairment (MCI), and AD, that is, across the complete AD spectrum [19]. In addition, this study showed that a lower fat-free mass, an important indicator of malnutrition [62], was associated with a higher risk of cognitive deterioration, with a comparable effect extent as BMI. Previous findings from the same group showed that patients with subjective cognitive decline (considered one of the earliest noticeable manifestations of AD and related dementias) reporting the lowest consumption of vegetables had the worst cognitive alterations [63]. Soysal et al., in a cross-sectional study, compared the nutritional status and micronutrient levels in outpatients with different types of dementia (AD, frontotemporal dementia, Lewy body dementia, vascular dementia, and normal pressure hydrocephalus) and found a prevalence of malnutrition of 17.2% and a risk of malnutrition in 43.2% of patients, according to the mini nutritional assessment [64].

A Korean study reported that among persons with an early diagnosis of AD, a reduction in cortical thickness was associated with an unhealthy and inadequate nutritional intake [65]. The course of dementia is very variable; diverse factors contribute to the uneven progression of cognitive, physical, and functional decline, with nutrition playing a crucial role [18]. Appropriate nutrition is essential for the proper functioning and repair of body systems, including the brain and the nervous system [12,66]. Inadequate nutrition is a frequent condition among older people and even more in residents of nursing homes [67], who are frequently affected by cognitive decline and dementia. It is confirmed that poor nutritional status is associated with adverse health outcomes such as increased hospitalizations, morbidity, and mortality [68]. Nutritional status seems to impact the prognosis of the progression of functional performance, cognitive decline [19], and behavioral disturbances [21]. A recent retrospective study [69] of the “PROtein enriched MEDiterranean diet to combat undernutrition and promote healthy neuroCOGnitive ageing” (PROMED-COG) project evaluated the association between undernutrition and cognitive decline and incident dementia in 9071 older adults (age range between 42 and 101 years) from three Italian population-based studies. The authors found a 14.3% prevalence of undernutrition at baseline, which was higher among women and in older participants, ranging from 3.5% in those aged <60 years to 28.8% in those aged >85 years. Undernutrition was associated with both incident cognitive decline and incident dementia over a median follow-up of 8.3 and 8.6 years, respectively [69]. These results highlight the importance of early identification of inadequate nutritional status because its management may be an important nonpharmacologic strategy to counteract neurodegeneration. These results highlight the need for an early detection of malnutrition in order to take action even in the early stages of the disease [70,71]. Other studies have shown an association between malnutrition, in particular weight loss, and cognitive decline in persons with dementia [72,73,74]. There is evidence that weight loss may precede the onset of AD [75,76,77], and older adults with lower BMI had worse cognitive deterioration and an increased risk of incident dementia [78,79]. Other studies have confirmed that low baseline BMI was associated with clinical progression after two years of follow-up in patients with MCI [80,81]. In our study, participants with AD and cognitively normal controls had a mean normal to slightly increased BMI, but persons with dementia had significantly lower adherence scores to the MedDiet, probably reflecting a poor-quality diet, which, besides BMI, as already discussed, may have important consequences from a cognitive and functional point of view. As a matter of fact, there was a significant correlation between adherence to the MedDiet and self-autonomy, as well as with cognitive decline. A study including MCI patients suggested that lower adherence to the MedDiet was associated with a greater risk of progression to AD [16]. The maintenance of functional status is a major goal in geriatric medicine because it is an essential component of the older adult’s health and quality of life. Functional loss is a common pathway to many chronic diseases associated with aging, and this is particularly true in patients with dementia [82]. Maintenance of functional status depends on several factors, some of which are modifiable, preventable, and reversible, including poor nutritional status [68] or poor-quality diet and lifestyle. Therefore, identifying these modifiable determinants in old age, especially for people with dementia, is crucial to help improve the quality of life and reduce disability and dependence [83].

According to widely recognized recommendations, malnutrition screening should be performed in all people with a diagnosis of dementia, administering food according to the person’s needs and preferences [17]. Appetite stimulants and oral nutritional supplements to correct cognitive impairment are not recommended. Conversely, ESPEN guidelines for nutrition and hydration in geriatrics recommend the use of oral nutritional supplements if needed in order to prevent protein–energy undernutrition in older adults [68].

We observed a remarkably reduced physical activity in patients with AD. The low levels of physical activity seen in patients with AD could be related to the progression of the disease that limits physical activity. It has been shown that exercise can help cognitive function in all phases of life, probably due to its effects on improving blood circulation and vascular function by reducing hypertension and increasing nitric oxide production, thus promoting neuronal brain plasticity and reducing neuroinflammation [84]. Data support exercise training to improve cognitive function in healthy older adults, while evidence in AD patients has not been strongly conclusive. This means that exercise seems to be useful for the management of AD at initial stages and for the prevention of incident dementia, but it is not yet defined, and no certain recommendations can be made about the specific exercise recommendations for AD prevention or treatment. More well-designed research is still needed to be able to recommend it with certainty. However, there is evidence that people who regularly exercise at a moderate to vigorous intensity on several days during the week have larger brains compared to those who do not exercise [85]. In addition, both physical and cognitive activity seem clearly related to better brain and cognitive resilience markers across cohorts with differing educational, racial, and disease statuses, supporting their potential neuroprotective effects [86], while studies in experimental animals have suggested that myokines secreted during exercise, such as irisin, may have neuroprotective actions [87].

According to the latest review by the *Lancet Commission* on dementia [3], there is evidence reporting that harmonized care and being actively engaged in diverse types of activities may contribute to reducing depression and neuropsychiatric symptoms and improve well-being in people with dementia, which may also have relevant benefits for caregivers. The success of such interventions has been related to personalizing the programs according to individuals’ interests, abilities, and preferences, and involving the family and carers. Few studies have evaluated the cost-effectiveness of these types of programs; hence, further investigations are needed in order to evaluate the requirements and feasibility of their implementation [3]. Conversely, RCTs of exercise in persons with dementia have been so far negative for mental health domain improvements in either care homes or communities [88,89].

A recent international collaborative guideline from European scientific societies and other stakeholders recommends considering physical activity for the primary prevention of dementia. The role of physical activity in slowing the progression from MCI to dementia is still uncertain; the greatest supporting evidence is that of mind–body interventions; the guideline recommends that exercise may be used for maintaining the functional ability and cognition in people with moderate dementia. Even if the scientific evidence on the beneficial effects of physical activity and exercise in the maintenance of cognitive functions in persons with normal cognition, MCI, or dementia comes from still inconclusive studies with very low or low certainty of evidence, the guideline recommends their application due to their beneficial effects on nearly all facets of health [90].

**Figure 3:**
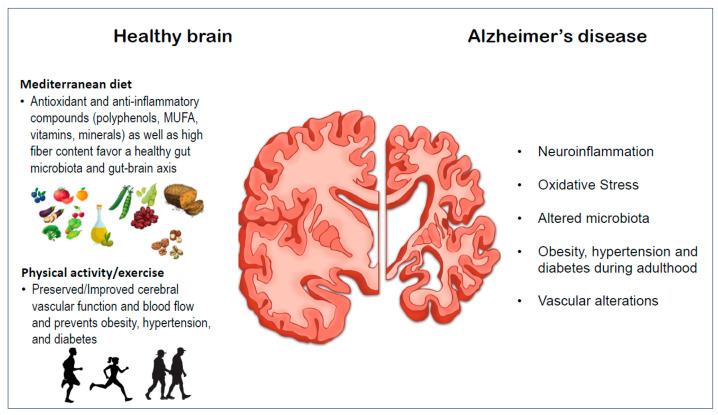
Some mechanisms that may help explain the neuroprotective effects of the Mediterranean diet and physical activity/exercise in the prevention of AD. MUFA: mono-unsaturated fatty acids.

Some of the mechanisms that may help explain the potential neuroprotective effects of the MedDiet and exercise include the reduction in neuroinflammation and oxidative stress, reduced insulin resistance, and improved vascular function and cerebral blood flow. Neuroinflammation has demonstrated a crucial role in the pathogenesis of AD [43]. Neuroinflammation is characterized by a primary immune reaction to brain injury mediated by key pro-inflammatory cytokines involving the activation and priming of glial cells, during which microglia exert macrophage-like functions such as vital surveillance, scavenging, antigen presentation, and cell repair [91]. The released cytokines increase blood–brain barrier permeability [92], which increases their own concentrations in the brain and intensifies microglia’s pro-inflammatory responses [93]. Several components of MedDiet exert antioxidant and anti-inflammatory actions that may contribute to attenuating the neuroinflammatory state [94,95]. Various polyphenols (e.g., tyrosol, anthocyanins, and isoflavones) have been found in extra-virgin olive oil, vegetables, fruits, nuts, and legumes and have demonstrated anti-inflammatory properties by modulating various pathways involved in oxidative stress, inflammatory mediators, and promoting cell survival mechanisms [96]. Moreover, the gut–brain axis has recently been proposed as a key player influencing multiple states or diseases, being susceptible to modification by factors such as diet [97]. Likewise, although physical activity and exercise are considered an essential strategy for the prevention and treatment of AD, their specific mechanisms for ameliorating AD are still not fully understood. Nevertheless, there is evidence highlighting their role in reducing neuroinflammatory responses even in the early stages [98] as well as a prominent role in the prevention of hypertension, diabetes, and the resulting cardiovascular damage [99], all recognized risk factors for developing AD (Figure 3).

The findings of our study should be interpreted within its limitations. First, it was conducted on a relatively small group of patients. When separating the groups by sex, the numbers were small and did not consent analyses comparing data from men and women; therefore, future studies are needed to include a larger number of participants from both sexes. Second, any cross-sectional study biases due to sampling, length-time bias, and residual confounding cannot be excluded, as well as the inability to establish a clear temporal association between exposure and outcome, preventing any conclusion of causality. Third, unfortunately, we did not have complete data on other crucial variables that may have an impact on cognitive decline and dementia, such as socioeconomic status, educational level, loneliness, and sleep quality/disorders. We also did not have information on other nutritional components that may be relevant to dementia risk, such as fiber, sweetened beverages, or ultra-processed food. Furthermore, age-matched case–control studies have several limitations, as indicated in two studies [100,101]. First, matching does not eliminate confounding; it may introduce selection bias if age is associated with exposure. Second, matching for age may distort dose–response relationships between age and outcomes. Finally, residual confounding requires complex adjustments and challenging interpretation. The strength of our study is that it is one of the few studies exploring the adherence of the high-quality Mediterranean diet and physical activity among patients who already have a diagnosis of Alzheimer’s disease.

## 5. Conclusions

Alzheimer’s disease patients had low adherence to the Mediterranean diet and very low levels of physical activity, reflecting unfavorable diet quality and lifestyle. Further attention is needed in these already compromised patients because of the possible implications of the increased risk of malnutrition and faster disease progression.

## Figures and Tables

**Table 1:** Descriptive characteristics in cases (affected by dementia) and controls.

Parameter	Cases (n = 73)	Controls (n = 73)	*p*-Value
***Demographics***			
Age (mean, SD)	76.6 (4.7)	76.3 (3.9)	0.69
Males (%)	42.5	20.5	0.004
Moderate-to-high physical activity level (%)	1.4	13.7	0.03
Low physical activity level (%)	38.4	93.2	<0.0001
Smoking status, no (%)	82.2	86.3	0.34
Smoking status, current (%)	5.5	6.8	
Smoking status, previous (%)	12.3	6.9	
*** Multidimensional evaluation***			
ADL (mean, SD)	3.4 (1.8)	6	-
IADL (mean, SD)	1.9 (2.1)	8 for women, 6 for men	-
MMSE (mean, SD)	16.7 (6.0)	30	-
Multimorbidity (%)	68.5	9.6	<0.0001
Polypharmacy (%)	74.0	28.8	<0.0001
***Nutritional evaluation***			
BMI (mean, SD)	26.5 (4.0)	26.1 (4.4)	0.54
Alcohol drinking, non-red wine (%)	5.5	0	0.12
Spices more than 3 times in a week (%)	32.9	49.3	0.04
Tea consumption more than 3 times in a week (%)	24.7	34.2	0.20
Cocoa, more than 3 times in a week (%)	23.3	13.7	0.21
Red fruits, more than 3 times in a week (%)	52.1	11.0	<0.0001
Coffee, yes/no (%)	72.6	100	<0.0001
Salt, 3 spoons/day (%)	4.1	0	<0.0001
MEDAS (mean, SD)	6.7 (1.4)	8.1 (1.5)	<0.0001

ADL: activities of daily living; BMI: body mass index; IADL: instrumental activities of daily living; MEDAS: Mediterranean Diet Adherence Screener; MMSE: mini-mental state examination; SD: standard deviation.

**Table 2:** Factors associated with moderate-to-high adherence to Mediterranean diet.

Parameter	Odds Ratio	95% Low CI	95% High CI	* p* -Value
Presence of Alzheimer’s Disease	0.222	0.058	0.848	0.028
Males	0.916	0.368	2.281	0.850
Age	0.929	0.845	1.022	0.129
Low physical activity level	0.905	0.345	2.375	0.839
Multimorbidity	0.762	0.238	2.443	0.648
Polypharmacy	0.930	0.297	2.917	0.902
Red fruits, more than 3 times per week	0.855	0.350	2.090	0.731
Coffee consumption	1.475	0.494	4.403	0.486
Salt, 2 spoons/day	0.587	0.232	10.484	0.260
Salt, 3 spoons/day	10.358	0.101	18.185	0.817

Data are reported as odds ratio (OR) with their 95% confidence intervals (CIs) and *p*-values, among factors statistically significant between cases (presence of dementia) and controls.

## Data Availability

The data and the databases are available upon reasonable request to the corresponding author.
